# Sensible Advice

**Published:** 1884-07

**Authors:** Charles Julius Hempel


Sensible Advice.—He who deviates from Hahnemann's principles in the treatment of chronic diseases lacks wisdom.—Charles Julius Hempel.

